# Hypokalemia after rituximab administration in nephrotic syndrome: two case reports

**DOI:** 10.1186/s12882-023-03079-4

**Published:** 2023-07-18

**Authors:** Yiyun Song, Lin Ding, Xin An, Yi Zhao, Xianhua Li, Xiangdong Yang, Xiaoyan Xiao

**Affiliations:** Department of Nephrology, Qilu Hospital of Shandong University, No. 107 Wenhua West Road, 250000 Jinan, Shandong PR China

**Keywords:** Rituximab, Hypokalemia, Nephrotic syndrome

## Abstract

Rituximab, a chimeric anti-CD20 monoclonal antibody, is an effective treatment for nephrotic syndrome. Hypokalemia is a rare adverse reaction among patients treated with rituximab although there have been extensive reports of acute and chronic adverse events with the administration of rituximab. We herein report two cases of symptomatic hypokalemia after intravenous rituximab administration in our center, to help health professionals consider the possibility of acute hypokalemia after rituximab administration, monitor potassium timely and develop an appropriate treatment plan.

## Introduction

Nephrotic syndrome (NS) is a group of diseases characterized by heavy proteinuria (proteinuria > 3-3.5 g/24hours), hypoalbuminemia (serum albumin < 30 g/L), edema, and hyperlipidemia. Approximately 80–90% NS cases in adults are idiopathic, caused by primary glomerular diseases such as primary membranous nephropathy (MN), focal segmental glomerulosclerosis (FSGS), minimal change disease (MCD), and IgA nephropathy [1, 2]. Specific immunosuppressive treatment plans should be formulated according to different causes.

Traditional immunosuppressive drugs for MN and MCD include glucocorticoid, cyclophosphamide and/or calcineurin inhibitors (CNIs). In recent years, new therapies with clear efficacy and less side effects, such as CD20-targeted therapy have been emerging.

Rituximab is a human-mouse chimeric anti-CD20 monoclonal antibody with B-cell-depleting effect. Rituximab is used to treat multiple hematological malignancies such as non-Hodgkin’s lymphoma (NHL) and chronic lymphocytic leukemia (CLL), and autoimmune diseases including rheumatoid arthritis [3, 4]. In the past decade, the application of rituximab in glomerular diseases has been rising, which provides more options for NS treatments [5]. Rituximab is recommended as a first-line treatment for MN and frequently relapsing/steroid-dependent MCD by the KDIGO 2021 Clinical Practice Guideline for the Management of Glomerular Diseases [6–8].

Numerous literatures have been reported on acute and chronic adverse events after rituximab administration. It is rarely reported that acute hypokalemia with clinical manifestations after rituximab administration.

## Case report

### Case 1

A 25-year-old young man was admitted to the Department of Nephrology, Qilu Hospital of Shandong University in June 2022 due to bilateral lower limb edema and proteinuria for 6 months. In December 2021, without obvious inducement, the man developed pitting edema of bilateral lower limbs with foam urine and no other symptoms such as gross hematuria, frequent urination, urgent urination, dysuria, fever, and backache. Then, he first sought care at the Department of Nephrology, Qilu Hospital of Shandong University. The examination showed that the level of his urinary protein was 3+, urinary protein-to-creatinine ratio (UPCR) was 3.36 g/g, serum albumin was 27.4 g/L, total cholesterol was 7.32 mmol/L, low-density lipoprotein cholesterol (LDL-C) was 5.02 mmol/L, and anti-PLA2R antibody was 22.7 RU/mL. The man with a positive anti-PLA2R antibody was diagnosed as MN without a renal biopsy [7]. Then he was given comprehensive treatment including blood pressure control with renin angiotensin system inhibitors (RASI), lipid regulation, and diuretic detumescence. The patient chose rituximab instead of conventional glucocorticoids and immunosuppressant therapy because of his personal preference.

**Fig. 1:**
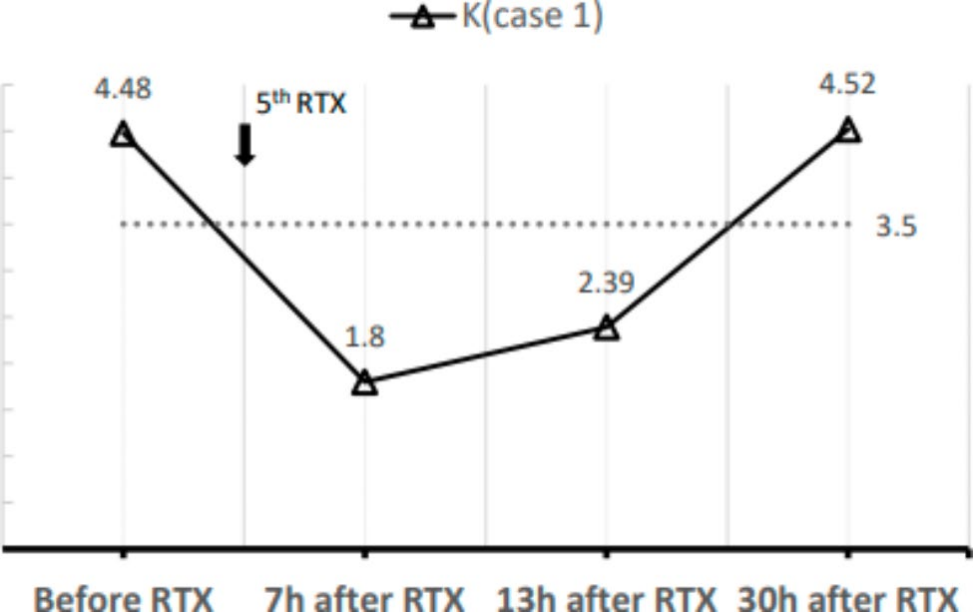
Potassium levels of case 1 (mmol/L) over time and laboratory normal value (3.5 mmol/L, dotted line). K, potassium (mmol/L); RTX, rituximab; h, hour

Since December 2021, the man was scheduled for his intravenous rituximab infusion of 500 mg weekly for 4 weeks, with total dose of 2000 mg. The man occurred anaphylaxis during the initial intravenous rituximab infusion, characterized by itchy scalp, facial and back rashes, which resolved with dexamethasone anti-allergy therapy, and he completed subsequent rituximab infusions. After 4 weeks of rituximab treatment, his disease received remission, with the count of CD19 + B cell decreased to 0 /uL, anti-PLA2R antibody turned negative, and proteinuria decreased. However, in June 2022, he was found that his disease relapsed, with increased proteinuria and CD19 + B cell. Therefore, the man was scheduled for his 5^th^ intravenous rituximab infusion (375 mg/m^2^, 500 mg daily for two consecutive days, total dose 1000 mg), in our Nephrology Unit in June 2022. Before starting the infusion, a venous blood test was performed: his serum potassium level was 4.48 mmol/L—sodium, calcium, magnesium, phosphorus, and glucose levels were normal. After 1.5 h of the infusion the man reported fatigue and the infusion was slowed down. About 7 h after the infusion ended, his asthenia became significantly worse, and even the man was unable to stand and move. A second venous blood test was then obtained, the potassium level was 1.8 mmol/L, and calcium (1.95 mmol/L), magnesium (0.6 mmol/L) and phosphorus (0.68 mmol/L) also decreased. Therefore, he was given intravenous potassium chloride injection (total dose K = 2 g) and oral potassium chloride solution (total dose K = 2 g). About 13 h after the infusion ended, the serum potassium level was 2.39 mmol/ L, he gained oral potassium chloride solution again (total dose K = 4 g). About 30 h after the infusion ended, his symptoms were alleviated, and his potassium level was returned to the normal range (4.52 mmol/L). At subsequent follow-ups, the patient serum potassium levels remained within normal limits. (shown in Fig. 1)

### Case 2

A 75-year-old man was admitted to the Department of Nephrology, Qilu Hospital of Shandong University in September 2022 due to bilateral lower limb edema and proteinuria for 2 months. In August 2022, without obvious inducement, the man developed pitting edema of bilateral lower limbs with foam urine, dark urine. Therefore, he sought care in our Nephrology Unit. The examination showed that the level of his urinary protein was 3+, UPCR was 2.25 g/g, and serum albumin was 41.3 g/L. Then, he found that the level of his UPCR increased to 14.78 g/g, and serum albumin decreased to 31.3 g/L. Therefore, he underwent renal biopsy during hospitalization in our department, and he was finally diagnosed as MCD. Because of the serious side effects of using adequate glucocorticoids in the elderly, the man finally received a combination treatment of RASI, lipid regulation, diuretics, reduced glucocorticoids (intravenous methylprednisolone, 40 mg/d, total 7days; oral prednisone 20 mg/d after discharge), and intravenous rituximab infusion.

**Fig. 2:**
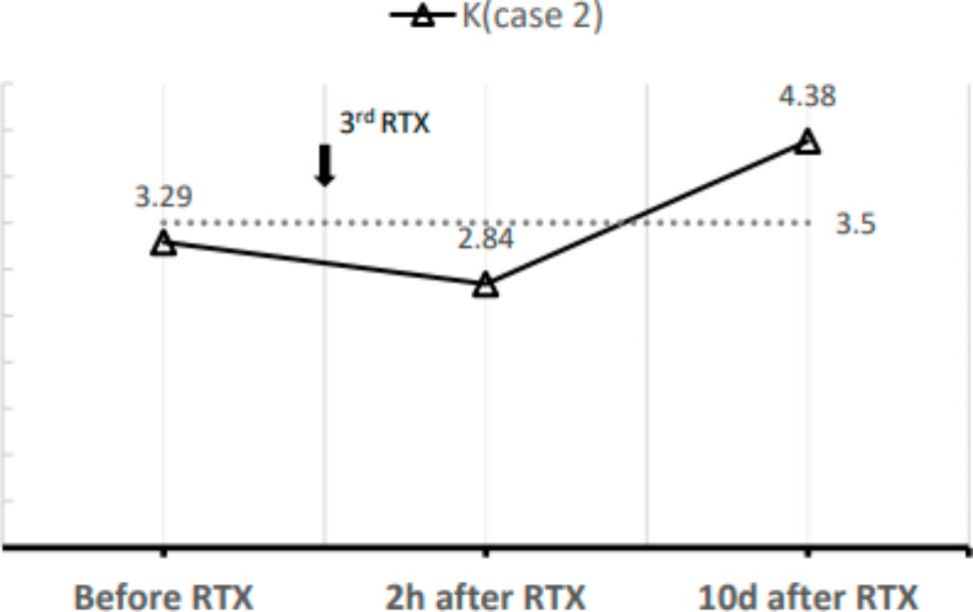
Potassium levels of case 2 (mmol/L) over time and laboratory normal value (3.5 mmol/L, dotted line). K, potassium (mmol/L); RTX, rituximab; h, hour; d, day

Since August 2022, the man was scheduled for his intravenous rituximab infusion (375 mg/m^2^ weekly). The man occurred anaphylaxis during the first rituximab treatment with a total dose of 500 mg, characterized by rashes and pruritus on the back, which relieved spontaneously. Almost a week later, he was infused with rituximab only 300 mg instead of 500 mg to prevent infection, without adverse reactions. Then, the man was scheduled for his 3^rd^ intravenous rituximab infusion (375 mg/m^2^, total dose 500 mg), in our Nephrology Unit in September 2022. Before starting the infusion, a venous blood test was performed: his serum potassium level was 3.29 mmol/L—sodium, calcium, magnesium, phosphorus, and glucose levels were normal before the infusion. He was given oral potassium chloride tablets 1 g, three times a day. After 2 h of the infusion, the man reported fatigue and lower limb muscle cramps, so the infusion was temporarily stopped. A second venous blood test was then obtained, the potassium level was 2.84 mmol/L, and calcium (2.09 mmol/L) and sodium (136 mmol/L) also decreased. He was given intravenous potassium chloride injection (total dose K = 0.75 g) in addition and intravenous calcium gluconate injection (total dose Ca = 1 g). His symptoms were resolved, and he completed subsequent rituximab infusions. At follow-ups, his serum potassium returned to a normal level. (shown in Fig. 2)

## Discussion

Biologics have emerged as an important modality of treatment in renal diseases and have allowed nephrologists to explore various new indications. Rituximab, a chimeric monoclonal antibody that targets the B-cell CD20 antigen, has been approved for the treatment of NS [3]. Although relatively safer than conventional medicines such as glucocorticoid and cyclophosphamide, rituximab is also observed to appear a few serious adverse events in practice. The most common side effects of rituximab intravenous administration are acute infusion-related reactions consisting of fever, chills, rash, and pruritus [9–11]. Other occasional reactions include infections, hypotension, hypertension, myocardial infarction, bronchospasm, and hypoproteinaemia [12–14].

Hypokalemia is a significant adverse event in hospitalized patients that may trigger cardiac arrhythmias and/or respiratory arrest. Only one case of hypokalemia caused by rituximab administration has been reported in the literature as yet [15]. To reduce prednisone dependence, a young woman diagnosed with idiopathic NS was started on rituximab. She felt dizziness and palpitation and was diagnosed with acute hypokalemia recognized by blood gas analysis when she was scheduled for her 6th intravenous rituximab infusion. Her symptoms were rapidly controlled by intravenous potassium administration. There were two patients developed symptoms of asthenia and were confirmed acute hypokalemia after rituximab infusion in our center. Similar acute reversible hypokalemia suggests that hypokalemia may be closely related to rituximab infusion.

It has been reported that hypokalemia is an adverse event of complex chemotherapy including rituximab for lymphoma treatment [16]. But none of these reports suggests hypokalemia is directly related to rituximab. Hypokalemia induced by platinum-containing drugs is secondary to hypomagnesemia [17]. Intracellular magnesium depletion reverts the inactivation of voltage-dependent renal outer medulla K channels (ROMK), thus increasing K secretion in the distal nephron [18]. Such hypokalemia may not be corrected by potassium supplementation until the hypomagnesemia is corrected. Abiraterone leads to the accumulation of mineralocorticoids, resulting in increased cortical collecting duct potassium secretion and ensuing hypokalemia [19, 20]. In addition, hypokalemia is also reported with trastuzumab, cetuximab, and lumretuzumab through drug-induced diarrhea [21–23]. However, hypokalemia has been reported as the most frequent electrolyte disorder after the administration of many anticancer-targeted therapies, but not rituximab [22]. The mechanism of rituximab leading to acute hypokalemia is unknown, which may affect potassium channels. It has been found that rituximab significantly decreased intracellular Ca^2+^ concentration and inhibited intermediate-conductance Ca^2+^-activated K (IK) channels [24]. In addition to complement-dependent cytotoxicity previously described, rituximab was also found to induce apoptosis of malignant B lymphocyte by stimulating FcγRIIB receptors and inhibiting Kv1.3 channels [25]. It has been reported that potassium calcium-activated channel subfamily N member 4 (KCNN4) channels are upregulated on the surface of B cells in patients with pemphigus treated with rituximab [26]. Nevertheless, KCNN4 channels promote the efflux of potassium, which is unlikely to cause hypokalemia.

Hypokalemia has a variety of etiologies and symptoms that are often ignored in hospitalized patients. Our patients in general had no signs and symptoms of discomfort, and their blood potassium level was normal in the previous days. However, blood potassium decreased rapidly after an intravenous infusion of rituximab. The acute, symptomatic, and rapidly reversible hypokalemia experienced by the patient indicates a close association with rituximab infusion. In addition, the patients did not take any medicine that would reduce blood potassium such as furosemide. Their magnesium and thyroid hormone levels were normal, both excluding a possible link with hypomagnesemia or hyperthyroidism. Previous individuals reported that nonspecific symptoms like dizziness and fatigue occurred after rituximab treatment. There may be related to undetected hypokalemia. More experimental data should be provided to clarify the relationship between rituximab and hypokalemia.

In conclusion, rituximab-based therapy is associated with a significant risk of hypokalemia. Early monitoring and effective management of hypokalemia are important for patients who receive rituximab -based therapy.

## Data Availability

All datasets presented in this study are included in the article/supplementary material.
